# Ultrarapid Multimode Microwave Synthesis of Nano/Submicron β-SiC

**DOI:** 10.3390/ma11020317

**Published:** 2018-02-22

**Authors:** Min Zhao, Michael Johnson, Wenzhi He, Guangming Li, Chen Zhao, Luling Yu, Juwen Huang, Haochen Zhu

**Affiliations:** 1 State Key Laboratory of Pollution Control and Resources Reuse, College of Environmental Science and Engineering, Tongji University, Shanghai 200092, China; zhaomin98971@126.com (M.Z.); zcyujyo@163.com (C.Z.); yululingyz@126.com (L.Y.); jwhuang@tongji.edu.cn (J.H.); haochen_zhu@tongji.edu.cn (H.Z.); 2 Shanghai Collaborative Innovation Centre for WEEE Recycling, Shanghai Second Polytechnic University, Shanghai 201209, China; 3 Department of Electronic & Computer Engineering, University of Limerick, Limerick V94 T9PX, Ireland; Michael.Johnson@ul.ie

**Keywords:** microwave, β-SiC, X-ray diffraction, refinement

## Abstract

This paper presents the design, development and realization of a fast and novel process for the synthesis of 3C silicon carbide (β-SiC) nanorods and submicron powder. Using SiO_2_ (or Si) and activated carbon (AC), this process allows β-SiC to be synthesized with almost 100% purity in timeframes of seconds or minutes using multimode microwave rotary tube reactors under open-air conditions. The synthesis temperature used was 1460 ± 50 °C for Si + AC and 1660 ± 50 °C for SiO_2_ + AC. The shortest β-SiC synthesis time achieved was about 20 s for Si + AC and 100 s for SiO_2_ + AC. This novel synthesis method allows for scaled-up flow processes in the rapid industrial-scale production of β-SiC, having advantages of time/energy saving and carbon dioxide emission reduction over comparable modern processes.

## 1. Introduction

In the material manufacturing sector, energy efficiency, sustainability and economic viability have become increasing important to the industry and society [1]. Recently, microwave heating has been found to offer faster, simpler and more cost-effective processes for material manufacture, with the result that it is now widely used for chemical synthesis, particularly for the preparation of novel functional materials [2,3,4,5]. One such material is Silicon carbide (SiC), an important ceramic material that is in high demand. Using current processes, the production of SiC is a costly undertaking, requiring large amounts of energy and high temperatures in addition to a lengthy synthesis time. Researchers have shown that microwave heating techniques may be adopted to allow SiC (itself an inorganic material with a strong microwave absorption rate) to be manufactured quickly and energy efficiently [1,5,6,7,8].

SiC is a compound with a 1:1 stoichiometric ratio of silicon and carbon, exhibiting many desirable physical and mechanical properties such as a high degree of hardness, high oxidation and corrosion resistance, low thermal expansion coefficient and high thermal conductivity [9,10]. More than 200 polymorphs of SiC have been identified thus far, with the cubic one of 3C-SiC being categorized as β-SiC and the hexagonal polytypes of 2H-, 4H- and 6H-SiC classified as α-SiC, based on different stacking sequences of the Si-C atomic layer [11,12,13]. β-SiC is widely used in many areas, such as electric vehicle production, nuclear energy, light-emitting diodes (LEDs), high-power electronic devices, electromagnetic wave shielding and absorbing applications.

SiC powders can be produced through industrial and laboratory methods. Using industrial methods, SiC powders are manufactured using the Acheson process through the carbothermic reduction of silicon dioxide (SiO_2_) using carbon powder at extremely high temperatures (2200–2400 °C) with a heating time of 30 h [4,9,11,14]. Using this approach, the resultant powder (consisting of α-SiC, β-SiC and other different crystalline phases of SiC) is usually of large particle size and consumes prodigious amounts of energy during the fabrication process [4]. This kind of SiC product is mainly used in low-tech applications (e.g., grinding and cutting), but applications with more stringent demands require SiC with a pure crystal form/nanostructure.

Nanomaterials have shown promising potential in many fields due to their improved optic, mechanic, electronic and magnetic properties compared to bulk materials [15,16]. For some time now, researchers worldwide have worked on the synthesis of nanometer SiC [17]. Diverse methods for the manufacturing of SiC nanoparticles have been reported in the literature, such as β-SiC nanoparticles [18], β-SiC films [19,20], β-SiC nanowhiskers [16], β-SiC nanorods [17], β-SiC nanofibers and β-SiC nanowires [21,22,23,24,25]. Chemical vapor deposition (CVD) is frequently used to synthetize SiC powder using a gaseous carbon or silicon source as precursors [18,26,27]. Other methods include sol-gel processes [28], combustion synthesis [29], thermal plasma [30,31,32], carbothermic reduction [4,33], microwave heating processes [1,6,10,16,23,34].

Two different types of microwave heating processes have been reported in the literature: single mode and multimode microwave heating. Advantages of single mode microwave heating include high energy density and fast heating speed, but its application in industry is limited because of the small resonant cavity [35]. For example, a 2450 MHz microwave single mode resonant cavity (waveguide is the resonant cavity) section size is typically only about 110 × 55 mm [35]. Multimode microwave heating employs a much larger application space and is widely used in household cooking, drying and other industrial fields with lower temperature requirements [11,35]. 

The research described in this paper sought to find a process for efficient β-SiC fabrication using the multimode microwave heating technique. Granular activated carbon (AC) and graphite were chosen as the microwave susceptor as both have a high microwave absorption rate and resist high temperatures. For the microwave device design and manufacturing, a rectangular multimode microwave cavity (RMMC) rotary reactor (e.g., a domestic microwave oven with improvements) was used for preliminary research and exploration. Using this platform, β-SiC synthesis was improved in line with industrial application requirements. Subsequently, an improved cylindrical multimode microwave cavity (CMMC) rotary reactor was designed and fabricated. This CMMC reactor had a higher microwave power rating than the RMMC reactor, facilitating greater time/energy savings. In addition, this “tunnel style” reactor allows production efficiency to be greatly improved, and furthermore enables the design of scaled-up flow processes using this device. In the future, flexible industrialized production of SiC can be realized using this design, helping to reduce stock and inventory requirements for manufacturers. “Tunnel style” microwave reactors can be used individually or in series as required, with the internal SiC production materials being laid out on a removable refractory material and the production materials moving though the production process.

## 2. Experimental

### 2.1. Materials and Devices

Powdered AC (chemically pure) and granular AC (c.p., 0.5–1 mm) were used as the reactant and microwave susceptor, respectively. Both materials were purchased from Sinopharm Chemical Reagent (SCRC) Co., Ltd. (Shanghai, China). Scale graphite (technical pure, compressed before use) was also used and was purchased from SCRC Co., Ltd. (Shanghai, China).

**Figure 1:**
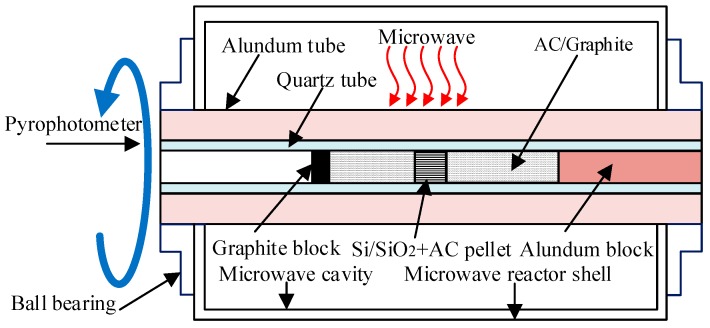
Reaction set-up for silicon carbide (SiC) synthesis in the rectangular multimode microwave cavity (RMMC) and cylindrical multimode microwave cavity (CMMC) reactors.

The prototype RMMC rotary reactor used in this research was modified from a microwave oven (having a single microwave tube, 800 W power input) with an alundum/quartz tube which was installed in the centre of the microwave oven, such that the tube could be rotated by a motor drive during the microwave heating. The resulting RMMC rotary reactor allowed for a more uniform microwave irradiation while enabling easy measurement of the temperature (via a pyrophotometer) and other parameters. The newly designed and manufactured CMMC rotary reactor, developed from this RMMC, is a reactor with a cylindrical multimode microwave cavity, having a larger scale and higher microwave power output (4 × 1.25 kW). A single alundum/quartz tube was installed in the centre of this CMMC reactor, with the tube again being rotated by a motor drive (as shown in Figure 1) during the fabrication process. The microwave frequency of both reactors was 2.45 GHz. 

### 2.2. SiC Synthesis Using RMMC and CMMC Reactors

For the fabrication of β-SiC using the RMMC and CMMC rotary reactors, stoichiometric amounts of Si and AC (1:1 molar ratio), SiO_2_ and AC (1:3 molar ratio) were added to a planetary ball mill which milled the materials for 7 h at a speed of 400 rpm. The milled powder was then cold-pressed into an 8 mm pellet die (5 tons), which was then mixed with distilled water to form a thick slurry before being pressed into individual pellets (0.3–0.35 g each). These pressed pellets (total weight of 0.6–0.7 g) were embedded in the granular AC/scale graphite with the microwave susceptor in the center of an open small quartz tube. Inside the quartz tube, a small cylindrical graphite block with several small grooves etched on the surface was placed on the left-hand side of the device for water vapor release during microwave sintering. On the right-hand side was a long movable alundum block, whose main function was the prevention of granular AC overflow during microwave sintering. The tube was rotated in the multimode microwave reactor using a motor during the microwave heating process (as shown in Figure 1). All preparations were performed at ambient pressure in open air. The conditions for all selected samples during these experiments are summarized in Table 1 (for the RMMC reactor) and Table 2 (for the CMMC reactor). The microwave sintering temperature was measured from light conducted by the quartz tube wall using an optical pyrophotometer (700/2000 °C; measurement error: 5.0%, Shanghai Automation Instrumentation Factory, WGG2-201) (Figure 1). 

### 2.3. Characterisation

The analysis of the fabricated samples from the RMMC/CMMC reactors was conducted using powder X-ray diffraction (XRD) (sourced from D8 Advance, Bruker, Karlsruhe, Germany with a Cu Ka1 radiation, λ = 1.54056 Å). Initial data were used to identify product phases with reference to the International Centre for Diffraction Data (ICDD) PDF database using the Jade 6.5 software (Materials Data Ltd., Livermore, CA, USA). Crystallographic parameters and quantitative phase fractions of crystalline components were obtained using the Rietveld refinement method (Topas 4.3) and RIR (Reference intensity ratio or K-value) method (Jade 6.5) against the XRD data (range of 10° < 2θ/° < 85° with a step size of 0.017°). Morphology investigation was performed by means of a scanning electron microscopy (SEM) (PHILIPS XL 30, Dutch Philips, Amsterdam, The Netherlands), allowing for the analysis of the microstructure of the SiC nanorods and submicron powder. Fourier Transform Infrared Spectoscopy (Nicolet 5700 FT-IR, Thermo Nicolet Corporation, Waltham, MA, USA) was conducted in the range of 400–4000 cm^−1^ with KBr plates for solid samples. Raman spectra were obtained using a laser Raman spectrometer (XploRA, Horiba Jobin Yvon, Paris, France) with a 532 nm laser used as the excitation source (power~80 mW, acquisition time 1 s). All Raman measurements were carried on at room temperature in the back-scattering geometry.

## 3. Results and Discussion

**Figure 2:**
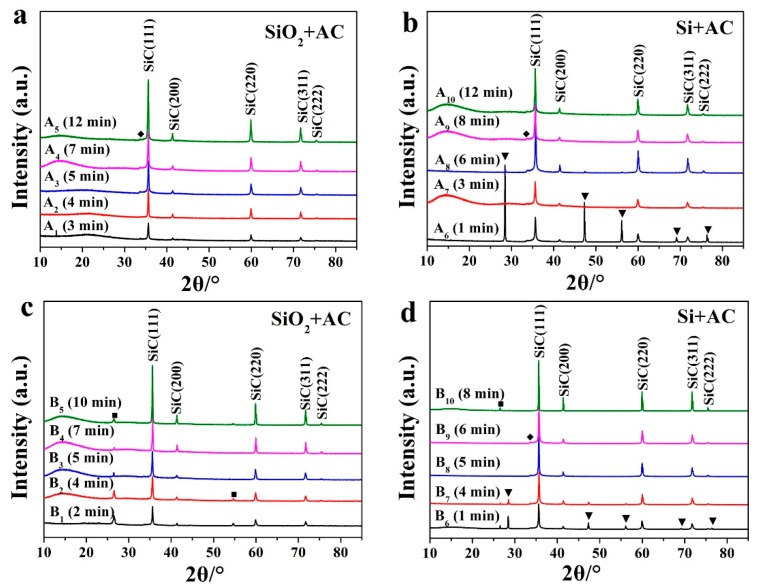
XRD patterns of β-SiC samples synthesized in RMMC reactor. Reflections from silicon (▼), graphite (■) and stacking faults in β-SiC (◆) are indicated. Microwave susceptor: (**a**,**b**) AC and (**c**,**d**) graphite.

Using microwave heating technologies, β-SiC powder was successfully synthesized in minutes using the RMMC rotary reactor and in seconds using the newly developed CMMC rotary reactor. For the RMMC rotary reactor, using SiO_2_ as the silicon source and granular AC or scale graphite as the microwave susceptor, β-SiC nanorods and submicron powder was synthesized in about 5–7 min. Figure 2 shows the XRD analysis results of this β-SiC (PDF card #75-0254) fabrication process for different heating times. As can be seen from the figure, the crystal structure of the resulting β-SiC was unaffected by heating time. A small peak (2θ = 33.6°) ahead of the highest intensity peak is assumed to be due to high-intensity stacking faults on the (111) plane in β-SiC, marked with (◆) [22,36,37].

**Figure 3:**
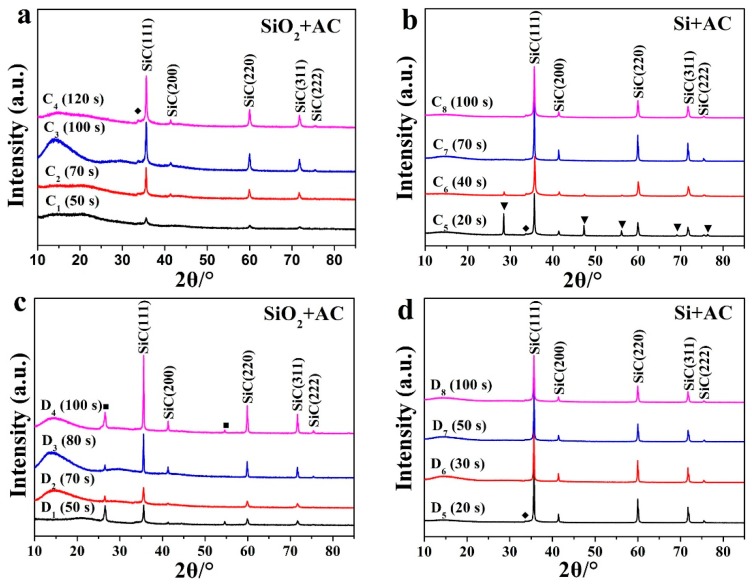
XRD patterns of β-SiC samples synthesized in CMMC reactor. Reflections from silicon (▼), graphite (■) and stacking faults in β-SiC (◆) are indicated. Microwave susceptor: (**a**,**b**) AC and (**c**,**d**) graphite.

**Figure 4:**
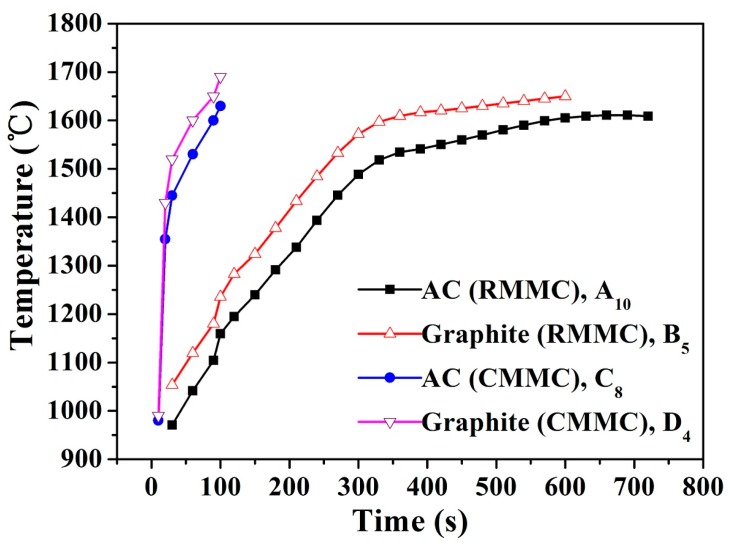
Heating curves of AC/graphite Microwave susceptor in RMMC and CMMC reactors.

Using the CMMC reactor and SiO_2_ as the silicon source, β-SiC formation was achieved after 50 s of heating (Figure 3, C_1_, D_1_). The intensity of the peaks was positively correlated with the temperature. Under the same heating conditions, the sintering temperature of the graphite used as the microwave susceptor is higher than that of activated carbon when used as the microwave susceptor (Figure 4). The intensity of the XRD peak in Figure 3c is higher than that in Figure 3a. The main reason for this is fact that the crystallinity of the synthesized SiC under high temperature conditions is better than that of the synthesized samples under low temperature. With the same microwave susceptor, the sintering temperature gradually increased with the increase of microwave heating time. The results indicate that in the same group, the intensities of the diffraction peaks show a gradually increasing trend mainly due to the reason discussed above.

The β-SiC absorption peaks in the XRD figures for this process are consistent with those reported in the literature [15,22,32,38,39,40]. Whether using the RMMC or the CMMC reactor, it was noted that a certain gas was generated and the resulting pellets always fractured. With regard to this reaction gas, the synthesis mechanism of SiC is relatively complex with SiO_2_ as the silicon source. The ideal synthesis reaction processes are shown as follows:

SiO_2_(s) + C → SiO(g) + CO(g) (1)

SiO(g) + 2C → SiC(s) + CO(g)
(2)

SiO(g) + 2CO→SiC(s) + CO_2_(g) (3)

**Figure 5:**
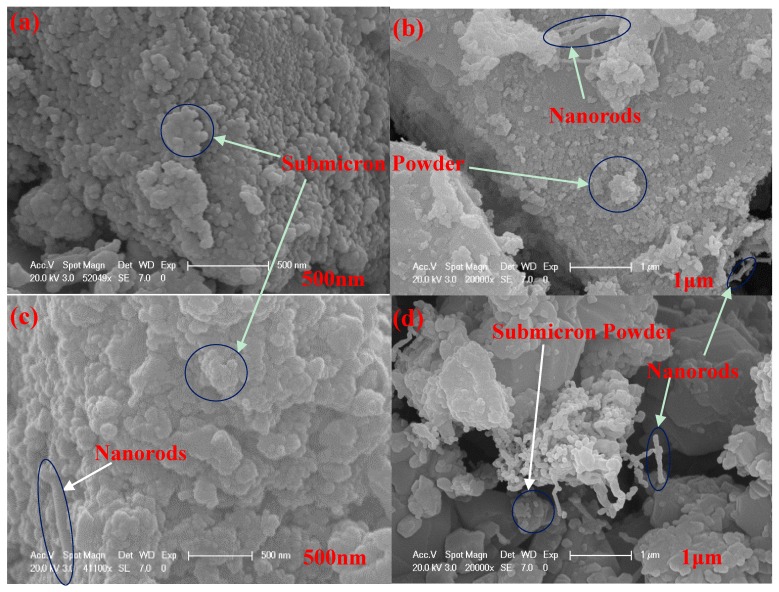
SEM micrographs of A_10_ (**a**); B_8_ (**b**); D_5_ (**c**); C_3_ (**d**), showing β-SiC nanorods and submicron powder.

The stoichiometric ratio of the raw materials is SiO_2_:C = 1:3. In the experiments, it was found that there was a small quantity of combustible gas present at the end of the quartz glass tube. This gas was found to be carbon monoxide (CO), from which it was deduced that the reactions (1) and (2) as described above dominate the β-SiC synthesis process. The product is a mixture of sub-micron β-SiC particles and nanorods (Figure 5d). At the beginning of the reaction, SiO_2_ and C particles are tightly bonded, the reaction between them is solid-solid reaction. The melting point and boiling point of SiO_2_ are 1650 ± 75°C and 2230 °C, respectively. The sublimation point of activated carbon is 3652 °C. In this study, the apparent temperature of most samples synthesized was 1600–1650 °C (Figure 4), with the highest apparent temperature of 1690 °C (Figure 4, D_4_), a temperature slightly below the melting point of SiO_2_. The generated SiO gas and C particles generate SiC particles through the reactions (1) and (2). With a continuous reaction, the binding force between SiO_2_ and C particles weakened gradually, while the generated SiC concurrently hinders the solid phase diffusion of C and the vapor diffusion of SiO. SiC can be produced from reaction (3) through the gas-gas exchange between SiO and CO, with the product being β-SiC nanorods (Figure 5d). The overall reaction is judged to be a solid-liquid-gas reaction. From the point-of-view that the product is a small amount of nanorods and more submicron β-SiC particles, (1) and (2) are the main reactions [38].

At the beginning of the reaction, AC is a kind of high-microwave energy absorbing material with a dielectric constant of about 5.8 and SiO_2_ is a kind of low-microwave energy absorbing material with a dielectric constant of 4.5. As the microwave irradiation time increases, a large amount of β-SiC (dielectric constant of 9.72) is generated and a large number of “hot spots” appeared in β-SiC of pellets. The number of these “hot points” of pellets is much higher than at the start of the microwave heating process, with the result that the sintering temperature increases rapidly and the overall rate of synthesis of β-SiC is accelerated. SiC synthesis is a strongly endothermic reaction, ΔH_298_ = 618.5 kJmol^−1^ [6] and the total reaction rate is directly related to the microwave input power and the amount of SiC generated during the reaction.

The successful preparation of the pellets is dependent on the moisture content of the material, as dry raw materials are not easily pressed into pellets and a small amount of distilled water is needed as a binding agent to facilitate the process. Through experimentation, it was discovered that the ideal moisture content for the raw material was 43–46%. If the water content is low, the viscosity of the raw material would be too low and the pellets would be too brittle (i.e., they would be easily cracked). Conversely, if the water content is too high, there will be too much water evaporation during the heating process, such that a large amount of energy will be absorbed by the water, thereby reducing the heating rate of the synthesized material. At the same time, the microwave radiation can also be absorbed by the excess water vapor, further lowering the heating rate of the reaction system. Therefore, the moisture content of raw materials is one of the key factors for the successful rapid synthesis of the SiC material.

In the RMMC reactor, with Si as the silicon source and granular AC as the microwave susceptor, the desired temperature for β-SiC synthesis was achieved in 1 min, showing part of β-SiC already formed (Figure 2, A_6_). At this point, there was still a high silicon diffraction peak in the sample A_6_ XRD graph, indicating that there was still some crystal silicon in the sample. This is attributed to “hot points”, a phenomenon common to microwave heating, similar to the effect achieved by focusing the sun's light through a refraction lens. These “hot points” reach the synthesis temperature first and the synthesis reaction begins here initially. By increasing the microwave heating time and the temperature of the whole sample, it was found that these diffraction peaks of Si disappeared, indicating that the reactions were complete (Figure 1, A_9_, A_10_). Under certain conditions, such as placing the pellets in the microwave focus area, more “hot points” will appear during the reaction and the temperature will rise rapidly, which results in a quicker reaction procedure and a shorter synthetic reaction time (Figure 2, A_8_; Figure 3, C_7_, D_5_, D_6_). From these results, it can be seen that temperature is another of the key factors for the efficient production of SiC. Through the optimized design of the microwave equipment used in these experiments, the area of this reaction microwave field is uniformly distributed such that the raw materials experience more uniform “hot spots”, resulting in a much improved SiC synthesis efficiency and quality.

With graphite as the microwave susceptor and a heating time of 1 min, there was only a small Si diffraction peak in the XRD graph of sample B_6_, B_7_, and this diffraction peak of elemental Si disappeared entirely after 5 min (Figure 2, B_8_–B_10_). The synthesis mechanism of SiC from Si and C was simple with only one step to complete the transformation, with no carbon gas being generated:

Si(l) + C → SiC(s)
(4)

The stoichiometric ratio of the raw materials is Si:C = 1:1. The content of β-SiC in the sample B_9_ was measured to be 100% (Figure 2d; Table 3, B_9_), in agreement with reaction (4) and with no carbon emitted directly.

The product is a mixture of sub-micron β-SiC particles and nanorods (Figure 5b,c). The melting point and boiling point of Si are 1410 °C and 2355 °C, respectively, and the sublimation point of AC is 3652 °C. The majority of the samples with better crystal structure were synthesized using reaction (4) at an apparent temperature of 1480–1610 °C (Figure 4) above the melting point of Si, with the overall reaction judged to be a solid-liquid reaction. At the beginning of the reaction, AC and silicon are high dielectric constant materials with dielectric constants of 5.8 and 11.9, respectively. Microwave heating produces a large number of “hot spots” and SiC begins to form in or near these “hot spots”. The precipitation of β-SiC crystal is caused by the change of local temperature and the change of carbon concentration in the liquid silicon. The solubility of carbon in liquid Si is less than 0.5%. Carbon is an endothermic process when dissolved in liquid silicon (Q_s_ = 247kJ/mol), but the crystallization of SiC from supersaturated solution is an exothermic process (crystallization heat Q_c_ = Q_R_ − Q_s_ = −362 kJ/mol). This means that the temperature of the C-Si interface tends to increase due to the dissolution and crystallization of the reaction system. As a result, the solubility of carbon increases, carbon diffuses from the high concentration of C-Si interface to a low concentration, with a very fast diffusion rate. When the temperature has reached its maximum, the solubility of the carbon and the concentration of carbon in the solution have also reached their highest values. As the temperature subsequently decreases, the dissolution of carbon in liquid silicon reaches supersaturation and the fluctuation of temperature will cause the precipitation of the β-SiC crystals. The dissolved atoms of carbon diffuse at the interface of the melt-carbon and the melt-silicon carbide and are “precipitated” from the melt by crystallization into β-SiC continuously. The β-SiC crystal precipitation, nucleation and growth rate are affected by the solubility of carbon in liquid silicon directly, so the total reaction rate of SiC synthesis is greatly affected by the solubility of the carbon in the liquid silicon solution [39].

In the CMMC reactor, using Si as the silicon source and AC as the microwave susceptor, the desired synthesis temperature for β-SiC was achieved in just 20 s. However, there was still residual Si in the pellets afterwards (as shown in Figure 3, C_5_). Increasing the heating time caused these diffraction peaks of crystal silicon to disappear (Figure 3, C_7_, C_8_). With graphite as the microwave susceptor, the reaction also completed in 20 s, with the content of β-SiC in the synthesized compound measured to be 100% (Figure 3, D_5_; Table 3, D_5_). These results coincide with observations from the experiments conducted by Carassiti regarding product synthesis in a single mode microwave reactor [40]. The β-SiC absorption peaks measured in the XRD figures compared favorably with those presented by other researchers [15,41].

The graphite diffraction peaks noted in B_1_–B_7_, B_10_ and D_1_–D_4_ result from the use of a graphite susceptor, as all the raw materials of the carbon source in the reaction is powdered AC. After the pellets are taken out from the graphite susceptor, graphite XRD diffraction peaks will appear in the results if the graphite on the pellet surface or the graphite mixed with pellet fragments is not removed completely.

In both the RMMC and CMMC reactors, when SiO_2_ was used as the silicon source, the synthesis process was found to be slower than when Si was used as the sole silicon source (Figure 2, A_1_, B_1_, B_6_), irrespective of whether granular AC or scale graphite was used as the microwave susceptor. This is due to the difference in bond energies between Si–O and Si–Si. The single bond energy of Si–O is 460 kJ, and the single bond energy of Si–Si is 196 kJ [42,43]. It is therefore more difficult to open the Si–O bond than the corresponding Si–Si bond under the same heating condition, ultimately leading to more time required in order to produce β-SiC using SiO_2_ as silicon source as opposed to using Si. The different bond energy results in different melting points (the melting point for SiO_2_ is 1660 ± 50 °C compared with the melting point of Si being 1410 °C [22,44]).

Figure 4 shows the heating curves of AC/graphite Microwave susceptor in the two reactors, as per the temperature data measured by the pyrophotometer. In the RMMC reactor, with the increase of microwave heating time, the temperature curve tended to gradually level out. In the CMMC reactor, initially the temperature rose quickly but subsequently slowed down as the heating time proceeded. Taking into account the temperature resistance of the quartz tube, the typical heating time is typically no more than 2 min. The temperature of the scale graphite used as the microwave susceptor was higher than that of AC by about 80 ± 15 °C for the same heating time, with the result that the compressed scale graphite is considered better than granular AC as the microwave susceptor. 

**Figure 6:**
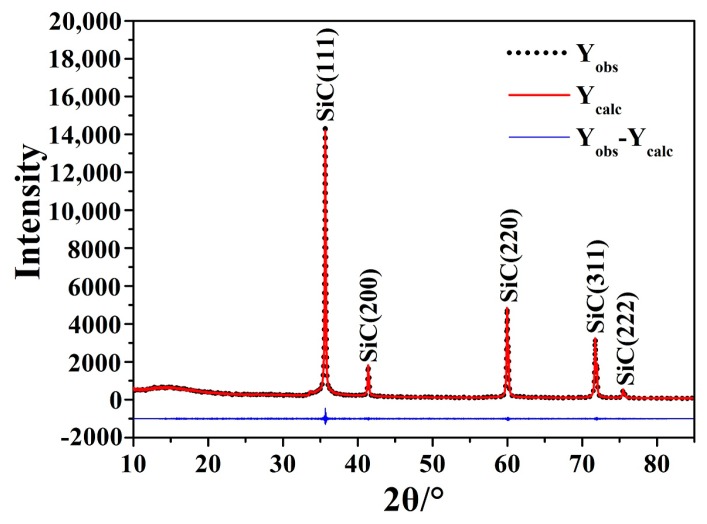
Profile plot for Rietveld refinement against XRD data of D_5_.

The samples A_8_, B_9_, B_10_, D_5_ were compared using the zinc blende structure of β-SiC as a starting model (Table 3). All structural modifications were merged and the basic parameters such as the corrected unit cell parameters could be obtained based on the D_5_ cell parameters (Table 3; Figure 6). 

Figure 5 shows the SEM graph structure of the β-SiC nanorods and submicron powder. The SEM image of the sample is similar to that reported by Kim et al. [45], and there was no obvious growth of the grains of the synthesized β-SiC product, with the submicron SiC product mainly coming from the grinding action. As can be seen from the figure, the sizes of the majority of the submicron powder were about 100 nm–1 μm with a uniform distribution (Figure 5a–d). A small amount of β-SiC nanorods were formed in B_8_ and C_3_ (Figure 5b,d) with diameters about 100 nm and length close to 1 μm. In all cases, the majority was β-SiC submicron powder as there were no other SiC crystal diffraction peaks (Figure 2 and Figure 3).

**Figure 7:**
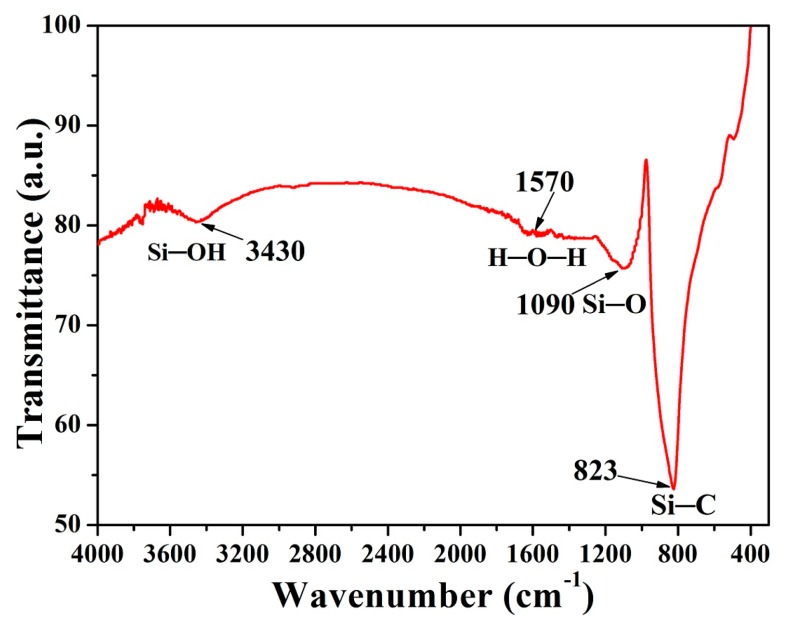
β-SiC FT-IR spectrum of A_8_.

Figure 7 shows the FT-IR spectrum of the A_8_ β-SiC submicro powder. The presence of an absorption peak at 823 cm^−1^ can be attributed to the Si–C stretching vibrations [30]. The broad peak at about 1090 cm^−1^ corresponds to the Si–O stretching vibrations [31,36,46], which is mainly due to the oxidation of the SiC surface. The amorphous SiO_2_ layer may be formed as part of the synthesis process (because the microwave susceptor contained tiny amounts of O_2_) or by the oxidation of SiC surface during the process of removing microwave susceptor at high temperature in air. The broad absorption band at around 3430 cm^−1^ shows a negligible Si–OH (silanol) peak, indicating that the surface modification causes the SiC surface to produce some silanols, which are relatively hydrophilic [47]. The peak at around 1570 cm^−1^ is due to the absorbed water [36,48]. Therefore, it can be inferred that the submicron powder produced consist mainly of SiC.

**Figure 8:**
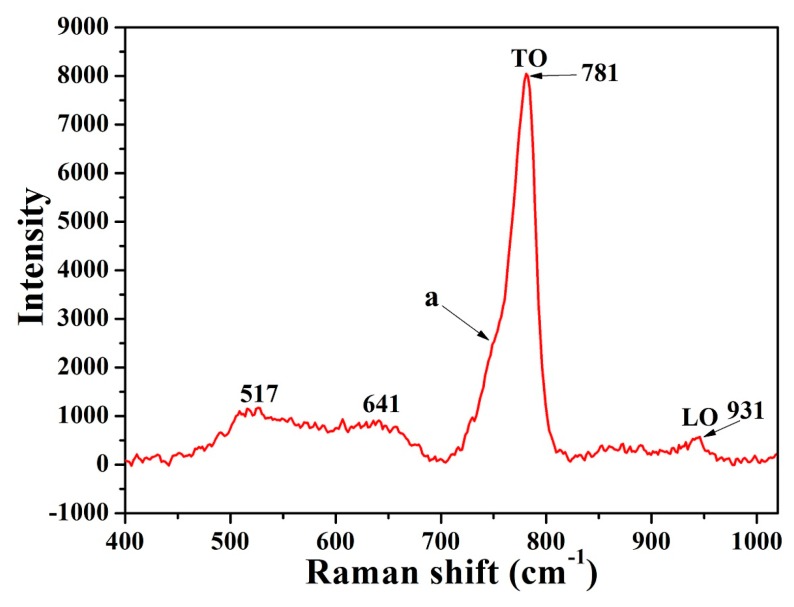
The Raman spectrum of D_5_.

The Raman spectrum of the β-SiC is shown in Figure 8. There were two optical phonon modes at the Г-point of the Brillouin zone in the Raman spectrum of β-SiC with zinc blende structure. There are two relatively sharp peaks located at 781 cm^−1^ and 931 cm^−1^ which can be attributed to the transverse optical (TO) phonon mode and longitudinal optic (LO) phonon mode of β-SiC, respectively. A shoulder (a point) on the strongest TO band (781 cm^−1^) could be attributed to TO (from points of the Brillouin zone other than the center) phonon mode scattering. Additional weaker bands at 517–641 cm^−1^ can be attributed to acoustic (transverse and longitudinal) phonon mode scattering [16,36,49,50].

## 4. Conclusions

This paper has presented a novel process for the fabrication of β-SiC, which produces material comparable to that of modern methods but at a fraction of the time, cost and energy required. Using two types of multimode microwave rotary tube reactors (with power levels of 800 W and 5000 W, respectively), with powdered AC, Si and SiO_2_ as the raw materials and utilizing granular AC or scale graphite as the microwave susceptor, high-purity β-SiC nanorods and submicron powder were synthesized successfully under open air conditions. The β-SiC samples were characterized with XRD and quantitatively analyzed using the crystal structure refine software. The results showed that the majority of the samples had 100% β-SiC content. The shortest synthesis time achieved in the experiments using this new process was between 20 s (Si + AC) and 100 s (SiO_2_ + AC). The resulting system and synthesis process is faster, more energy efficient and is greatly simplified compared to the current industrial standards. Experimentation has shown that scale graphite is preferable to granular AC as the microwave susceptor. The moisture content of the raw materials is an important factor, with the optimal content found to be 43–46%. Temperature was also found to be a critical factor to realize the rapid synthesis of β-SiC. The effects of variations in microwave heating time have been found to be minimal, but the presence of “hot points” is of importance for the initialization of the synthesis reaction. Therefore, it is important to design the microwave reactor to optimize these “hot points”, enabling more microwaves to focus in a specific area, such that the product can be produced quickly in this area. The reaction temperature was measured using light conducted by the quartz tube wall, solving the problem of microwave sintering sample temperature measurements. 

Experiments have shown that the optimal fabrication temperature was 1660 ± 50 °C (SiO_2_ + AC) and 1460 ± 50 °C (Si + AC), close to the melting point of the two silicon materials. The fabrication temperatures are lower than the boiling point of silica/silicon (2230/2355 °C) or the carbon sublimation point (3652 °C), indicating that the synthesis mechanism of β-SiC are liquid-solid (Si + AC) and vapor-liquid-solid reactions (SiO_2_ + AC). The research also provides a feasible technical solution for the recovery and recycling of waste crystalline silicon (e.g., waste solar cell). Furthermore, the use of this novel process can enable the design of scaled-up flow processes for rapid industrial-scale production of β-SiC and other new materials.

## Figures and Tables

**Table 1:** Selected RMMC samples and reaction conditions.

**Sample Number**		**A_1_**	**A_2_**	**A_3_**	**A_4_**	**A_5_**	**A_6_**	**A_7_**	**A_8_**	**A_9_**	**A_10_**
a	Silicon source	SiO_2_	SiO_2_	SiO_2_	SiO_2_	SiO_2_	Si	Si	Si	Si	Si
	Irradiation time (min)	3	4	5	7	12	1	3	6	8	12
**Sample Number**		**B_1_**	**B_2_**	**B_3_**	**B_4_**	**B_5_**	**B_6_**	**B_7_**	**B_8_**	**B_9_**	**B_10_**
b	Silicon source	SiO_2_	SiO_2_	SiO_2_	SiO_2_	SiO_2_	Si	Si	Si	Si	Si
	Irradiation time (min)	2	4	5	7	10	1	4	5	6	8

Carbon source: AC; radiation power: 800 W; Microwave susceptor: a: AC/b: graphite; atmosphere: Air.

**Table 2:** Selected CMMC samples and reaction conditions.

**Sample Number**		**C_1_**	**C_2_**	**C_3_**	**C_4_**	**C_5_**	**C_6_**	**C_7_**	**C_8_**
c	Silicon source	SiO_2_	SiO_2_	SiO_2_	SiO_2_	Si	Si	Si	Si
	Irradiation time (s)	50	70	100	120	20	40	70	100
**Sample Number**		**D_1_**	**D_2_**	**D_3_**	**D_4_**	**D_5_**	**D_6_**	**D_7_**	**D_8_**
d	Silicon source	SiO_2_	SiO_2_	SiO_2_	SiO_2_	Si	Si	Si	Si
	Irradiation time (s)	50	70	80	100	20	30	50	100

Carbon source: AC; radiation power: 5000 W; Microwave susceptor: c: AC/d: graphite; atmosphere: Air.

**Table 3:** Crystallographic data from Rietveld refinements against XRD data.

Sample	A_8_	B_9_	B_10_	D_5_
Phases, wt %	β-SiC: 98.1(6.3)%; Si: 1.9(0.1)%;	β-SiC: 100(7.5)%;	β-SiC: 95.4(4.3)%; Graphite: 4.6(0.2)%	β-SiC: 100(7.4)%;
Cell formula units/Z	4	4	4	4
α-Parameter/Å	4.3556	4.3584	4.3602	4.3600
Unit cell vol/Å^3^	82.63	82.79	82.89	82.88
Calculated density, ρ/g cm^−3^	3.233	3.217	3.213	3.213
Residue factor/R	18.66	18.98	19.69	17.77
Residue factor/R_p_	2.52	3.07	2.73	2.57
Residue factor/R_wp_	3.68	4.53	4.16	3.81
Residue factor/R_exp_	3.14	4.40	3.77	3.41

Space group: Cubic, F4-3m (2, 1, 6); Si 4a (0, 0, 0), C 4c (1/4, 1/4, 1/4).

## References

[1] Kitchen H.J., Vallance S.R., Kennedy J.L., Tapia-Ruiz N., Carassiti L., Harrison A., Whittaker A.G., Drysdale T.D., Kingman S.W., Gregory D.H. (2014). Modern microwave methods in solid state inorganic materials chemistry: From fundamentals to manufacturing. Chem. Rev..

[2] Schwenke A.M., Stumpf S., Hoeppener S., Schubert U.S. (2014). Free-standing carbon nanofibrous films prepared by a fast microwave-assisted synthesis process. Adv. Funct. Mater..

[3] Kennedy J.L., Drysdale T.D., Gregory D.H. (2015). Rapid, energy-efficient synthesis of the layered carbide, Al_4_C_3_. Green Chem..

[4] Birkel A., Lee Y.G., Koll D., Meerbeek X.V., Frank S., Choi M.J., Kang Y.S., Char K.H., Tremel W.F. (2012). Highly efficient and stable dye-sensitized solar cells based on SnO_2_ nanocrystals prepared by microwave-assisted synthesis. Energy Environ. Sci..

[5] Wang H., Zhu W.C., Liu Y.C., Zeng L.K., Sun L.Y. (2016). The Microwave-Assisted Green Synthesis of TiC Powders. Materials.

[6] Moshtaghioun B.M., Poyato R., Cumbrera F.L., de Bernardi-Martin S., Monshi A., Abbasi M.H., Karimzadeh F., Dominguez-Rodriguez A. (2012). Rapid carbothermic synthesis of silicon carbide nano powders by using microwave heating. J. Eur. Ceram. Soc..

[7] Van Laar J.H., Slabber J.F.M., Meyer J.P., van der Walt I.J., Puts G.J., Crouse P.L. (2015). Microwave-plasma synthesis of nano-sized silicon carbide at atmospheric pressure. Ceram. Int..

[8] Zhao M., Johnson M., He W.Z., Li G.M., Zhao C., Huang J.W., Zhu H.C. (2017). Transformation of waste crystalline silicon into submicro β-SiC by multimode microwave sintering with low carbon emissions. Powder Technol..

[9] Omidi Z., Ghasemi A., Bakhshi S.R. (2015). Synthesis and characterization of SiC ultrafine particles by means of sol-gel and carbothermal reduction methods. Ceram. Int..

[10] Moskovskikh D.O., Song Y., Rouvimov S., Rogachev A.S., Mukasyan A.S. (2016). Silicon carbide ceramics: Mechanical activation, combustion and spark plasma sintering. Ceram. Int..

[11] Kuang J.L., Cao W.B., Elder S. (2013). Synthesis of α-SiC particles at 1200 °C by microwave heating. Powder Technol..

[12] Nam D.H., Kim B.-G., Yoon J.-Y., Lee M.-H., Seo W.-S., Jeong S.-M., Yang C.-W., Lee W.-J. (2014). High-Temperature Chemical Vapor Deposition for SiC Single Crystal Bulk Growth Using Tetramethylsilane as a Precursor. Cryst. Growth Des..

[13] Aldalbahi A., Li E., Rivera M., Velazquez R., Altalhi T., Peng X.Y., Feng P.X. (2016). A new approach for fabrications of SiC based photodetectors. Sci. Rep..

[14] Pushpakaran B.N., Subburaj A.S., Bayne S.B., Mookken J. (2016). Impact of silicon carbide semiconductor technology in Photovoltaic Energy System. Renew. Sustain. Energy Rev..

[15] Seo Y.-K., Eom J.-H., Kim Y.-W. (2018). Process-tolerant pressureless-sintered silicon carbide ceramics with alumina-yttria-calcia-strontia. J. Eur. Ceram. Soc..

[16] Chen J.H., Liu W.N., Yang T., Lin B., Su J.D., Hou X.M., Chou K.C. (2014). A Facile Synthesis of a Three-Dimensional Flexible 3C-SiC Sponge and Its Wettability. Cryst. Growth Des..

[17] Xie W., Möbus G., Zhang S.W. (2011). Molten salt synthesis of silicon carbide nanorods using carbon nanotubes as templates. J. Mater. Chem..

[18] Wang Y., Zhang L., Zhang X.Y., Zhang Z.Z., Tong Y.C., Li F.Y., Wu J.C.S., Wang X.X. (2017). Open mouthed β-SiC hollow-sphere with highly photocatalytic activity for reduction of CO_2_ with H_2_O. Appl. Catal. B.

[19] Pawbake A., Mayabadi A., Waykar R., Kulkarni R., Jadhavar A., Waman V., Parmar J., Bhattacharyya S., Ma Y.R., Devan R. (2016). Growth of boron doped hydrogenated nanocrystalline cubic silicon carbide (3C-SiC) films by Hot Wire-CVD. Mater. Res. Bull..

[20] Wang S.H., Wang T., Druzhinin S.I., Wesner D., Jiang X., Schönherr H. (2017). Detailed Study of BSA Adsorption on Micro-and Nanocrystalline Diamond/β-SiC Composite Gradient Films by Time-Resolved Fluorescence Microscopy. Langmuir.

[21] Zhang M., Zhao J., Li Z.J., Yu H.Y., Wang Y.Q., Meng A., Li Q.D. (2016). Bamboo-like 3C-SiC nanowires with periodical fluctuating diameter: Homogeneous synthesis, synergistic growth mechanism, and their luminescence properties. J. Solid State Chem..

[22] Mukasyan A.S., Rogachev A.S. (2017). Combustion synthesis: Mechanically induced nanostructured materials. J. Mater. Sci..

[23] Wang P., Cheng L.F., Zhang Y.N., Zhang L.T. (2017). Synthesis of SiC nano bers with superior electromagnetic wave absorption performance by electrospinning. J. Alloys Compd..

[24] Chen J.J., Ding L.J., Xin L.P., Zeng F., Chen J. (2017). Thermochemistry and growth mechanism of SiC nanowires. J. Solid State Chem..

[25] Kang P.C., Zhang B., Wu G.H., Gou H.S., Chen G.Q., Jiang L.T., Mula S.H. (2014). Synthesis of β-SiC nanowires by ball milled nanoparticles of silicon and carbon. J. Alloys Compd..

[26] Yazdanfar M., Pedersen H., Sukkaew P., Ivanov I.G., Danielsson Ö., Kordina O., Janzén E. (2014). On the use of methane as a carbon precursor in Chemical Vapor Deposition of silicon carbide. J. Cryst. Growth.

[27] Zhang S., Xu Q.F., Tu R., Goto T., Zhang L.M. (2015). Growth Mechanism and Defects of <111>-Oriented β-SiC Films Deposited by Laser Chemical Vapor Deposition. J. Am. Ceram. Soc..

[28] Su J.J., Gao B., Chen Z.D., Fu J.J., An W., Peng X., Zhang X.M., Wang L., Huo K.F., Chu P.K. (2016). Large-Scale Synthesis and Mechanism of β-SiC Nanoparticles from Rice Husks by Low-Temperature Magnesiothermic Reduction. ACS Sustain. Chem. Eng..

[29] Agathopoulos S. (2012). Combustion synthesis of ultra-fine SiC powders in low pressure N_2_-atmosphere. Ceram. Int..

[30] Meng G.W., Zhang L.D., Qin Y., Mo C.M., Phillipp F. (1999). Synthesisof β-SiC nanowires with SiO_2_ wrappers. Nanostruct. Mater..

[31] Meng A., Li Z.J., Zhang J.L., Gao L., Li H.J. (2007). Synthesis and Raman scattering of β-SiC/SiO_2_ core-shell nanowires. J. Cryst. Growth.

[32] Seo Y.-K., KIM Y.-W., Nishimura T., Seo W.-K. (2017). High thermal conductivity of spark plasma sintered silicon carbide ceramics with yttria and scandia. J. Am. Ceram. Soc..

[33] Chabi S., Rocha V.G., Tuñón E.G., Ferraro C., Saiz E., Xia Y.D., Zhu Y.Q. (2016). Ultralight, Strong, Three-Dimensional SiC Structures. ACS Nano.

[34] Wang J.K., Zhang Y.Z., Li J.Y., Zhang H.J., Song S.P., Zhang S.W. (2017). Catalytic effect of cobalt on microwave synthesis of β-SiC powder. Powder Technol..

[35] Jin H.Q., Dai S.S., Huang K.M. (1999). Microwave Chemistry.

[36] Zhang J., Liu X.H., Jia Q.L., Huang J.T., Zhang S.W. (2016). Novel synthesis of ultra-long single crystalline β-SiC nanofibers with strong blue/green luminescent properties. Ceram. Int..

[37] Zhang M. (2017). Quasi-monodisperse β-SiC nanospheres: Synthesis and application in chemical-mechanical polishing. J. Phys. Chem. Solids.

[38] Wang F., Cao W.B., Sun J.L., He R.L. (2008). Synthesis and Preparation of SiC Powders at Low Temperature. Mater. Rev..

[39] Wang Y.X., Tan S.H., Jiang D.L. (2004). Research and Development of Reaction Sintered Silicon Carbide. J. Inorg. Mater..

[40] Carassiti L., Jones A., Harrison P., Dobson P.S., Kingman S., MacLaren I., Gregory D.H. (2011). Ultra-rapid, sustainable and selective synthesis of silicon carbide powders and nanomaterials via microwave heating. Energy Environ. Sci..

[41] Li Z.B., Wang Y.G., An L.N. (2017). Control of the thermal conductivity of SiC by modifying the polymer precursor. J. Eur. Ceram. Soc..

[42] Fritz G., Matern E. (1986). Carbosilanes: Syntheses and Reactions.

[43] Wu R.B., Yang G.Y., Pan Y., Chen J.J., Zhai R., Wu L.L., Lin J. (2008). Prism-shaped SiC nanowhiskers. J. Alloys Compd..

[44] Kumagai T., Izumi S., Hara S., Sakai S. (2007). Development of bond-order potentials that can reproduce the elastic constants and melting point of silicon for classical molecular dynamics simulation. Comput. Mater. Sci..

[45] Kim Y.-W., Lee Y.-I., Mitomo M. (2006). Sinterability of Nano-Sized Silicon Carbide Powders. J. Ceram. Soc. Jpn..

[46] Vix-Guterl C., Alix I., Gibot P., Ehrburger P. (2003). Formation of tubular silicon carbide from a carbonesilica material by using a reactive replica technique: Infra-red characterization. Appl. Surf. Sci..

[47] Zhou L.J., Huang Y., Xie Z.P. (2000). Gelcasting of concentrated aqueous silicon carbide suspension. J. Eur. Ceram. Soc..

[48] Zhang J., Li W., Jia Q.L., Lin L.X., Huang J.T., Zhang S.W. (2015). Molten salt assisted synthesis of 3C–SiC nanowire and its photoluminescence properties. Ceram. Int..

[49] Wu R.B., Zhou K., Yue C.Y., Wei J., Pan Y. (2015). Recent progress in synthesis, properties and potential applications of SiC nanomaterials. Prog. Mater. Sci..

[50] Yang K., Yang Y., Lin Z.M., Li J.T., Du J.S. (2007). Mechanical-activation-assisted combustion synthesis of SiC powders with polytetrafluoroethylene as promoter. Mater. Res. Bull..

